# A Case of Myxedema Coma in a 48-Year-Old Female Presenting With Altered Mental Status Post-trauma

**DOI:** 10.7759/cureus.97516

**Published:** 2025-11-22

**Authors:** Rohini Bhuvaneshwari Gurumoorthy, Learned Gonzales

**Affiliations:** 1 Family Medicine, Desert Regional Medical Center, Palm Springs, USA; 2 Critical Care Medicine, Desert Regional Medical Center, Palm Springs, USA

**Keywords:** altered mental status evaluation, clinical hypothyroidism, endocrine disorders, episodic hypothermia, hypothyroid myxedema coma, icd infection, icthyosis, myxedema crisis, post trauma

## Abstract

Myxedema coma is a life-threatening endocrine emergency characterized by altered mental status, hypothermia, and multi-organ system dysfunction. It often arises from long-standing, severe hypothyroidism and can be precipitated by acute stressors like trauma, infection, myocardial infarction, or surgery. Given the high mortality rate, immediate treatment with thyroid hormone replacement and stress doses of hydrocortisone (until coexisting adrenal insufficiency is ruled out) must be initiated immediately upon suspicion, before laboratory confirmation. In this report, we present the case of a 48-year-old female with altered mental status following an in-hospital fall that caused a left lower leg fracture during an outside admission for cellulitis. The patient had a strong family history of thyroid disorders and exhibited diffuse, global ichthyosis (a skin change) upon presentation. Following immediate treatment, all symptoms, including the ichthyosis, dramatically improved. This case highlights the importance of considering myxedema coma in patients with suggestive history and presentation, even when symptoms are precipitated by acute trauma.

## Introduction

Myxedema coma is a rare, life-threatening complication of severe hypothyroidism, most often affecting older adults and women, with an estimated incidence of 2.56 cases per one million US persons per year and a mortality rate ranging from 6.8% to 30% depending on the setting and definition [1,2]. It results from decompensation of longstanding hypothyroidism, often triggered by infection, cold exposure, stroke, myocardial infarction, or sedative drugs, leading to multi-organ dysfunction [1,3,4]. Clinically, patients present with hypothermia, altered mental status (confusion, stupor, coma), hypoventilation, bradycardia, hypotension, hyponatremia, hypoglycemia, and lactic acidosis, with rapid progression to respiratory and cardiovascular failure if untreated [1,2,5]. The differential diagnosis includes sepsis, adrenal insufficiency, stroke, and drug overdose, as these conditions can mimic the presentation [6,7].

Diagnosis is clinical but confirmed by laboratory findings of elevated thyroid-stimulating hormone (TSH) and low free T4 (primary hypothyroidism), or low free T4 with inappropriately normal/low TSH (secondary hypothyroidism), along with assessment of metabolic derangements and exclusion of adrenal insufficiency [1,6,7]. Management requires immediate intravenous levothyroxine and empiric glucocorticoids to address possible adrenal insufficiency, with liothyronine considered in critically ill patients; intensive supportive care for respiratory, cardiovascular, and metabolic complications is essential [1,5]. Complications include high mortality due to multiorgan failure, with delayed recognition and treatment significantly worsening outcomes [1,2,4]. It is vital to recognize that many patients do not exhibit overt myxedema or a comatose state; thus, altered mental status remains the most significant diagnostic clue. Patients require intensive care unit (ICU) admission for comprehensive pulmonary and cardiovascular support to manage this severe condition effectively.

## Case presentation

A 48-year-old female was transferred to our facility following a fractured left lower leg, sustained from an in-hospital fall at an outside institution where she had been admitted for cellulitis. She underwent an open reduction and internal fixation (ORIF) for the fracture. Prior to her planned discharge, the patient experienced a sudden and profound neurological decline, characterized by altered mental status and slurred speech. Her orientation fluctuated significantly, at times being alert and oriented to self only (ANO x 1), and at other times, disoriented to all four spheres (ANO x 4). A comprehensive stroke protocol was initiated, including extensive computed tomography (CT) imaging of the head and neck, which was unremarkable for any intracranial or cerebrovascular pathology. Her past medical history was notable for a chronic rheumatic disease causing global xerosis cutis (diffuse ichthyosis on physical exam). Despite being alert and oriented to self and capable of repeating her own name, she exhibited severe comprehension deficits to basic questions during bedside assessment.

Upon initial evaluation, the patient appeared ill and visibly uncomfortable. Physical examination revealed normocephalic, atraumatic head and neck with moist mucous membranes. Cardiovascular exam showed regular rate and rhythm without murmurs, gallops, or rubs. Pulmonary assessment demonstrated equal bilateral breath sounds with no adventitious sounds. The abdominal exam showed soft, non-distended, non-tender abdomen with normoactive bowel sounds. Extremities showed no clubbing, cyanosis, or edema. Her skin exhibited diffuse, global ichthyosis. Neurologically, she was able to move all extremities, but remained acutely altered, oriented only to self.

Initial laboratory findings from the day of transfer included a complete blood count (CBC) (Table 1) with microcytic anemia (hemoglobin 9.0 g/dL, hematocrit 31.9%, mean corpuscular volume (MCV) 71.0 fL, mean corpuscular hemoglobin (MCH) 20.1 pg, mean corpuscular hemoglobin concentration (MCHC) 28.3 g/dL, red cell distribution width (RDW) 25.5% (High)), and neutrophilia (neutrophil rel 84.2% (High), lymphocyte rel 13.3% (Low)). Comprehensive metabolic panel (Table 2) revealed hypernatremia (sodium 142.0 mmol/L (High)), hyperchloremia (chloride 112.0 mmol/L (High)), elevated blood urea nitrogen (BUN, 32.0 mg/dL (High)), low calcium (calcium 7.1 mg/dL (Low)), and an anion gap of 6. Thyroid function tests (Table 3) showed acute hypothyroidism with a low free thyroxine (T4) (0.56 ng/dL) and significantly elevated TSH (11.80 μIU/mL).

**Table 1:** Hematology Basic Lab Results Hgb: hemoglobin, Hct: hematocrit, MCH: mean corpuscular hemoglobin, MCV: mean corpuscular volume, MCHC: mean corpuscular hemoglobin concentration, MPV: mean platelet volume, RDW: red cell distribution width

Lab Test	Result	Normal Range
WBC	8.3 (10e^9^/L)	4.2-10.8 (10e^9^/L)
Hgb	9.0 (g/dl) (Low)	12-17 (g/dl)
MCV	71.0 (fl) (Low)	81-100 (fl)
MCHC	28.3 (%) (Low)	32-35.5 (%)
Platelet	327.0 (10e^9^/L)	150-410 (10e^9^/L)
RBC	4.500 (10e^12/^L)	4.2-5.6 (10e^12/^L)
Hct	31.9 (%) (Low)	38-50(%)
MCH	20.1 (pg) (Low)	26-35 (pg)
RDW	25.5 (%) (High)	12-18 (%)
MPV	8.4 (fl)	6.4-11 (fl)
Lymphocyte Rel	13.3 (%) (Low)	20-40 (%)
Eosinophil Rel	0.2 (%)	0-6 (%)
Neutrophil Rel	84.2 (%) (High)	42-77 (%)
Monocyte Rel	1.8 (%)	0-8 (%)
Basophil Rel	0.5 (%)	0-2 (%)

**Table 2:** Chemistry Comprehensive BUN: blood urea nitrogen

Lab Test	Result	Normal Range
Sodium Lvl	142.0 (mEq/l) (High)	133-141 (mEq/l)
Chloride Lvl	112.0 (mEq/l) (High)	98-107 (mEq/l)
Glucose Level	76 (mg/dl)	80-115 (mg/dl)
Creatinine Lvl	1.0 (mg/dl)	0.7-1.3 (mg/dl)
BUN/Creat	32.0 (no unit)	6.0-30.0 (no unit)
Potassium Lvl	4.6 (mEq/l)	3.4-5.0 (mEq/l)
CO2	24.0 (mEq/l)	21-30 (mEq/l)
BUN	32.0 (mg/dl) (High)	9.0-20 (mg/dl)
Anion Gap	6 (mEq/l)	6-13 (mEq/l)
Calcium Lvl	7.1 (mg/dl) (Low)	7.8-9.8 (mg/dl)

**Table 3:** Thyroid Panel TSH: thyroid-stimulating hormone

Lab Test	Result	Normal Range
T3 Free	3.04 (pg/ml)	2.0-4.4 (pg/ml)
TSH	11.80 (mIU/L) (High)	0.46-4.68 (mIU/L)
T4 Free	0.56 (ng/dl) (Low)	0.78-2.12 (ng/dl)

A comprehensive timeline detailing the patient's clinical progression and therapeutic interventions is provided below.

Day 1 (following transfer)

The patient was admitted to the ICU due to the acute change in mental status. Over the course of the day, she experienced significant fluctuations in blood pressure, ranging from extreme hypertension during the day to profound hypotension at night, ultimately requiring three vasopressor agents which included norepinephrine titrated to a max dose of 30 mcg/min, followed by vasopressin (dose of 0.03 units/min to max dose of 0.04 units/min) and epinephrine (dose of 5 mcg/min to a max of 30 mcg/min) and arterial line placement for hemodynamic monitoring [1]. She developed copious hypersalivary secretions, which were refractory to prolonged suctioning efforts, necessitating emergent intubation for airway protection. Despite aggressive warming measures, including a Bair Hugger and multiple blankets, she remained persistently hypothermic in the range of 30 to 35 Celsius for the entirety of Day 1. A detailed family history was elicited from her sister, revealing a history of thyroid disorder in the patient, Hashimoto's thyroiditis in her sister, and a rare form of lupus and rheumatoid arthritis in her mother, who was managed by a rheumatologist. Given the constellation of symptoms [1], myxedema coma was highly suspected, and appropriate evidence-based treatment [1,2-6] was initiated with hydrocortisone 100 mg IV push, Synthroid 50 mcg, and Triostat 5 mcg.

Day 2 (post-admission)

On Day 2, a "whiteout" of the left lung was noted on chest imaging, prompting a bronchoscopy due to potential mucous plugging. Bronchoscopy revealed and successfully cleared a significant amount of phlegm and mucus plugs from the patient's left main bronchus and segmental airways. Prior to this intervention, her vital signs showed low blood pressure, high heart rate and respiratory rate which normalized after bronchoscopy. She became normothermic, suggesting the effectiveness of the thyroid hormone replacement therapy. Serological studies were pending, and the patient's overall condition showed improvement, though she remained intubated. Neurology consultation opined that the change in mental status from Glasgow Coma Scale (GCS) 12 to 7 was likely due to global hypoperfusion secondary to worsening hypotension and possible metabolic encephalopathy, rather than acute ischemic stroke, given the lack of focal neurological deficits and negative imaging.

Day 3 (post-admission)

The patient's hemoglobin dropped from 9 g/dl to 5 g/dl, necessitating two units of packed red blood cell transfusion. After the first unit of red blood cell transfusion, hemoglobin improved. Enoxaparin was held, and sequential compression devices (SCDs) were initiated. A magnetic resonance imaging (MRI) brain and an electroencephalogram (EEG) were ordered to further evaluate her altered mental status. Additional crucial history was provided by the sister, who reported that their mother had a similar autoimmune disorder managed with steroids and gammaglobulin infusions every three months, beginning in her late 50s. The sister also disclosed that the patient had recent hospitalizations (approximately one month prior) at another facility for anemia, hypokalemia, and cellulitis. The EEG revealed moderate to severe diffuse slowing and the presence of triphasic waves, indicative of diffuse metabolic cerebral dysfunction and encephalopathy, supporting the clinical picture of a systemic metabolic derangement.

Days 4 to 5 (post-admission)

The patient's distributive shock began to slowly resolve with mean arterial pressure (MAP) >65 mm Hg, systolic blood pressure (SBP) >90 mm Hg. By the fifth day, she began responding to commands, was conscious and acknowledging questions but remained intubated. Her acute kidney injury with creatinine of 2.5 mg/dl resolved to normal levels of 1.0 mg/dl with treatment. The hydrocortisone taper was initiated which involved changing IV hydrocortisone 100 mg every eight hours to IV hydrocortisone of 50 mg every six hours. Sputum cultures returned positive for extended-spectrum beta-lactamase (ESBL) Klebsiella, and meropenem 1 g every 12 hours was initiated with a seven-day hard stop.

Days 6 to 7 (post-admission)

**Figure 1:**
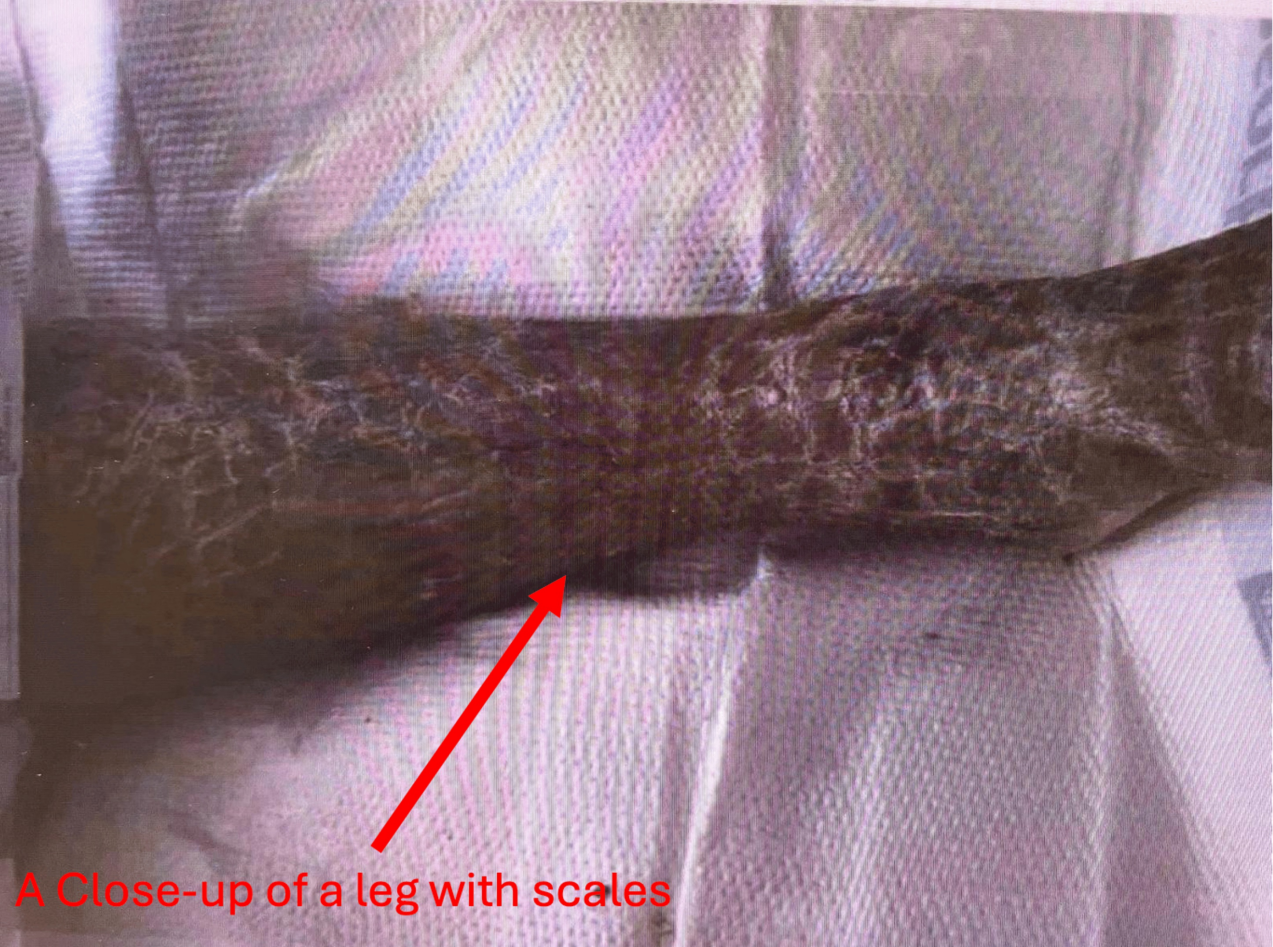
Appearance before treatment- Ichthyosis of skin seen in the leg

**Figure 2:**
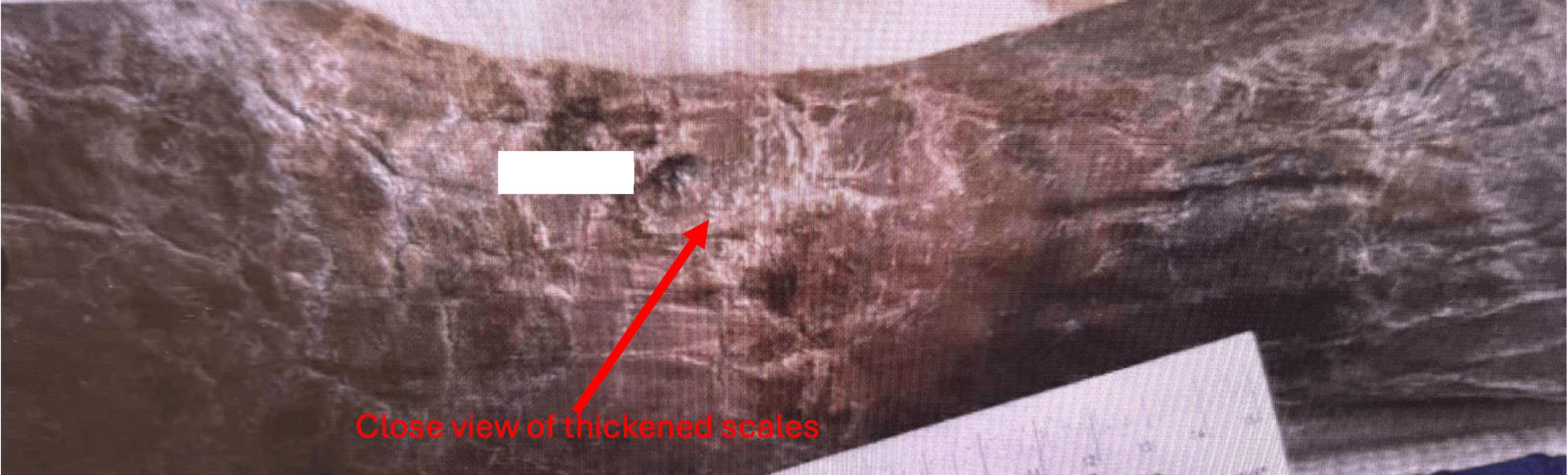
Before treatment, the appearance of the leg in closer view

**Figure 3:**
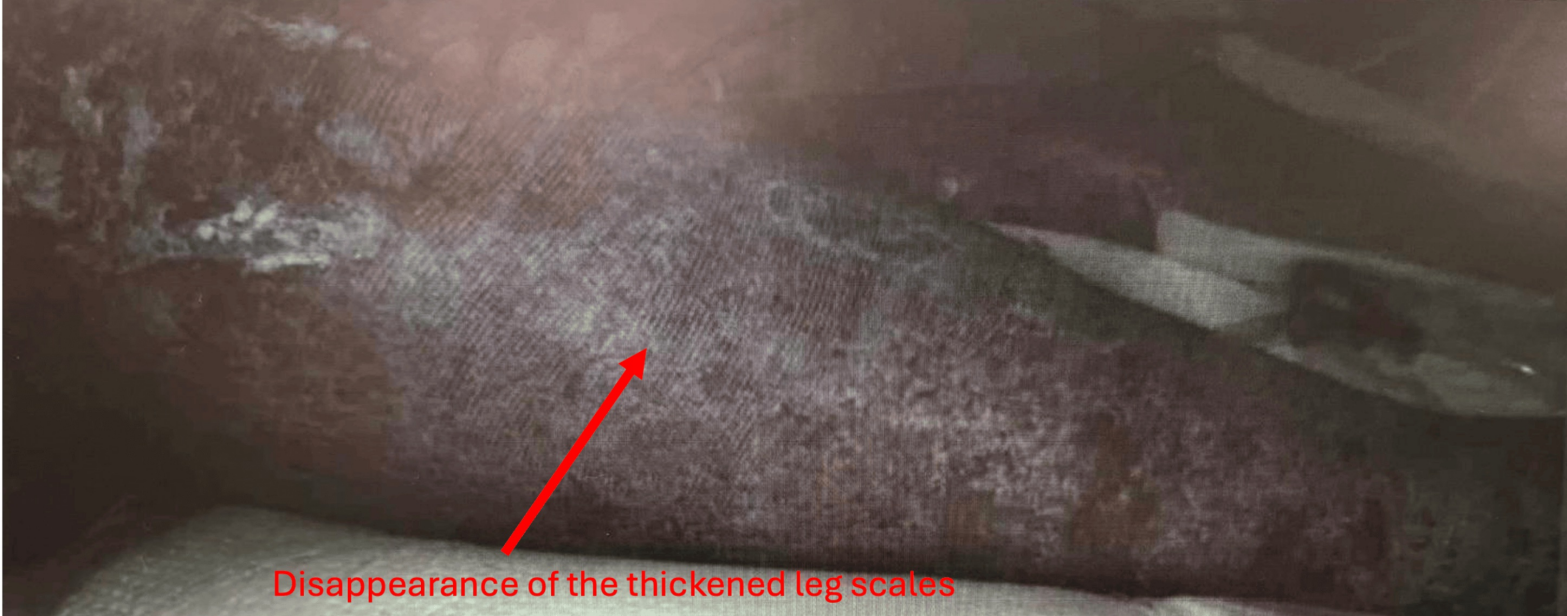
Post treatment appearance: scales in the healed skin are less prominent with ichthyosis slowly improving.

The patient's condition continued to improve. She was successfully extubated on the sixth day, transitioned to a regular diet, and became alert and oriented to all four spheres. She was aware of the reasons for her recent critical illness, denied pain or anxiety, and appeared comfortable. Her skin which showed global ichthyosis (a skin change) upon presentation is shown in Figure 1 and Figure 2. Thickened scales which were seen before the treatment gradually resolved and became less prominent after treatment which is shown in Figure 3.

Post-ICU course and discharge planning (following ICU stay)

Following her downgrade from the ICU, the patient's clinical course continued to show significant improvement. Infectious Diseases was consulted and recommended discontinuing meropenem, given the patient's clinical stability and the possibility of previous symptoms being secondary to a drug-induced hypersensitivity reaction, which was supported by the development of eosinophilia (absolute eosinophil count of 1.3*109/litre post-ICU Day 2). Further viral and parasitic studies (Coccidioides negative, Strongyloides, cytomegalovirus (CMV), Epstein-Barr virus (EBV) pending) were sent to investigate potential underlying etiologies for the eosinophilia. 

Her microcytic anemia was attributed to iron deficiency, evidenced by an iron panel (from early in her stay) showing low iron (<28 μg/dL), low ferritin (<11 ng/mL), and an iron saturation >223%. She started on ferrous sulfate 325 mg orally daily. Elevated liver enzymes, initially noted as transaminitis, were improving, and an abdominal ultrasound and hepatitis panel were performed, with the hepatitis panel returning negative.

The myxedema, secondary to severe hypothyroidism (TSH 11.80 on admission, improving to 9.29 the following day), resolved with levothyroxine therapy. Her medication was transitioned from 50 mcg IV twice daily to 88 mcg by mouth daily, with a plan to recheck TSH and free T4 approximately one week later. Acute respiratory distress secondary to increased secretions resolved following intubation and subsequent extubation. Leukocytosis, hypernatremia, hyperkalemia, and acute kidney injury (AKI) all resolved, with creatinine normalizing. Intermittent bradycardia also improved with thyroid hormone replacement. 

## Discussion

The presented case demonstrates a classic profile of myxedema coma, closely matching the epidemiology and clinical features described in the literature [1-3,8-12]. The patient’s features - older age, female sex, winter seasonality, and a strong family history of thyroid disease - are consistent with large cohort analyses and retrospective studies [1-3,9,12]. Clinical presentation in the case which includes acute mental status change, severe hypothermia, bradycardia, hypotension, and multi-organ dysfunction mirrors the cardinal features of myxedema coma [1-14]. The rapid progression to shock requiring vasopressors, hypoventilation, and metabolic derangements (hyponatremia, hypoglycemia) is well-documented in both reviews and case series [1,3-9,11-13]. The presence of a precipitating factor (infection) and a history of autoimmune thyroid disease further support the diagnosis [1,3-9,11-13].

Management in this case with immediate administration of intravenous hydrocortisone, levothyroxine, and liothyronine, along with intensive supportive care aligns with current guidelines [1,3,4,6,10,11]. The American Thyroid Association recommends empiric glucocorticoids to cover possible adrenal insufficiency, followed by intravenous levothyroxine as first-line therapy, with liothyronine considered in critically ill patients [1,3,4,6,10]. 

Early diagnosis and prompt hormone replacement are critical for survival, and clinical improvement is typically seen within days of therapy initiation [1,3-6,11]. Complications observed in this case, which include respiratory failure, infection, acute kidney injury, and anemia, are common in myxedema coma and contribute to its high mortality [1-3,8,9,11-14]. The patient’s rapid improvement in temperature and neurological status, resolution of shock, and eventual extubation are consistent with published data on recovery trajectories in the ICU setting [1-3,8,9,11-14]. 

Alternative differential diagnoses were considered and excluded based on clinical and laboratory findings [1,3,4,6,10,11,15]. Sepsis, adrenal crisis, hypoglycemia, and other causes of coma were ruled out by the constellation of hypothermia, bradycardia, metabolic derangements, and a strong history of hypothyroidism [1,3,4,6,10,11,15]. Thyroid storm was excluded by the absence of hyperthermia and tachyarrhythmias [15]. Adrenal crisis typically presents with hypotension and hypoglycemia but lacks the profound hypothermia and bradycardia seen here [1-4,6,10,11]. Neurological causes (stroke, drug overdose) were excluded by EEG findings of metabolic encephalopathy and the absence of focal deficits or toxic exposures [3,4,6,10,11,15]. The rapid clinical response to thyroid hormone and steroid therapy further supports myxedema coma as the diagnosis [1,3,4,6,10,11,15]. 

## Conclusions

This case highlights the challenges in diagnosing myxedema coma, particularly when masked by other acute medical conditions such as trauma and infection. The patient's rapid neurological decline and hemodynamic instability, in the absence of cerebrovascular pathology, alongside significant endocrine and metabolic derangements and a compelling family history of autoimmune and thyroid disorders, strongly suggested myxedema coma. Prompt recognition and initiation of appropriate thyroid hormone replacement and supportive care, including mechanical ventilation for airway protection and vasopressors for hemodynamic support, were critical in achieving a positive outcome. The case was further complicated by infectious processes, electrolyte abnormalities, anemia, and concerns regarding social vulnerabilities, all of which required multidisciplinary management. This report underscores the importance of a high index of suspicion for myxedema coma in patients with unexplained altered mental status, even in seemingly unrelated clinical contexts, and the necessity of comprehensive, integrated care for complex medical presentations.

## References

[1] Chaker L, Papaleontiou M (2025). Hypothyroidism: a review. JAMA.

[2] Chen DH, Hurtado CR, Chang P, Zakher M, Angell TE (2024). Clinical features and outcomes of myxedema coma in patients hospitalized for hypothyroidism: analysis of the United States National Inpatient Sample. Thyroid.

[3] Wartofsky L (2006). Myxedema coma. Endocrinol Metab Clin North Am.

[4] Kruithoff ML, Gigliotti BJ (2025). Thyroid emergencies: a narrative review. Endocr Pract.

[5] Bridwell RE, Willis GC, Gottlieb M, Koyfman A, Long B (2021). Decompensated hypothyroidism: a review for the emergency clinician. Am J Emerg Med.

[6] Wall CR (2000). Myxedema coma: diagnosis and treatment. Am Fam Physician.

[7] Ito H, Fukuda K, Ashida K (2022). Case report: myxedema coma caused by immunoglobulin A vasculitis in a patient with severe hypothyroidism. Front Immunol.

[8] Bourcier S, Coutrot M, Ferré A (2023). Critically ill severe hypothyroidism: a retrospective multicenter cohort study. Ann Intensive Care.

[9] Dutta P, Bhansali A, Masoodi SR, Bhadada S, Sharma N, Rajput R (2008). Predictors of outcome in myxoedema coma: a study from a tertiary care centre. Crit Care.

[10] Jonklaas J, Bianco AC, Bauer AJ (2014). Guidelines for the treatment of hypothyroidism: prepared by the American Thyroid Association Task Force on thyroid hormone replacement. Thyroid.

[11] Ueda K, Kiyota A, Tsuchida M, Okazaki M, Ozaki N (2019). Successful treatment of myxedema coma with a combination of levothyroxine and liothyronine. Endocr J.

[12] Taylor PN, Medici MM, Hubalewska-Dydejczyk A, Boelaert K (2024). Hypothyroidism. Lancet.

[13] McDermott MT (2020). Hypothyroidism. Ann Intern Med.

[14] Kaneko M, Ohara K, Shikata H (2022). A case of fatal myxedema coma with electrocardiogram Osborne J-wave in a patient initially diagnosed with hypothyroidism. Endocr J.

[15] Edlow JA, Rabinstein A, Traub SJ, Wijdicks EF (2014). Diagnosis of reversible causes of coma. Lancet.

